# Experimental investigation and multi-performance optimization of the leachate recirculation based sustainable landfills using Taguchi approach and an integrated MCDM method

**DOI:** 10.1038/s41598-023-45885-8

**Published:** 2023-11-04

**Authors:** Osama Khan, Sameera Mufazzal, Ahmad F. Sherwani, Zahid A. Khan, Mohd Parvez, Mohammad Javed Idrisi

**Affiliations:** 1 Department of Mechanical Engineering, Jamia Millia Islamia, New Delhi, 110025 India; 2 Department of Mechanical Engineering, Al Falah University, Faridabad, Haryana 121004 India; 3 Department of Mathematics, College of Natural and Computational Science, Mizan-Tepi University, Tepi, Ethiopia

**Keywords:** Ecology, Environmental sciences

## Abstract

Landfill leachates contain harmful substances viz. chemicals, heavy metals, and pathogens, that pose a threat to human health and the environment. Unattended leachate can also cause ground water contamination, soil pollution and air pollution. This study focuses on management of leachate, by recirculating the rich, nutrient-filled fluid back into the landfills, turning it to a bioreactor, thereby maximising the performance parameters of landfills favourable for electricity production by the waste to energy plants. This study demonstrates a sustainable alternative method for utilising the fluid, rather than treating it using an extremely expensive treatment process. Further, it also experimentally investigates the effect of varying levels of five input parameters of the landfill including waste particle size, waste addition, inorganic content in waste, leachate recirculation rate, and landfill age, each at five levels, on the multiple performance of the landfill using Taguchi’s L25 standard orthogonal array. Experimental results are analysed using an integrated MCDM approach i.e. MEREC-PIV method and statistical techniques such as analysis of mean (ANOM) and analysis of variance (ANOVA). The results indicate that the optimal setting of the input parameters is waste particle size at 9 ppm, waste addition at 80 Ktoe, inorganic content in waste at 2%, leachate recirculation rate at 250 l/day and landfill age at 3 years. Further, inorganic content waste is found to be the most significant parameter for the multiple performance of the landfill. This study presents a novel approach to produce input parameters for power plants which may enhance their profitability and sustainability.

## Introduction

Landfills are known to generate substantial quantity of heat due to decomposition of the organic waste material leading to rise in ambient temperature which may contribute to the overall global warming exacerbating the effects of climate change^1^. India, with its rapidly growing population, has seen an exponential growth in waste generation primarily due to rapid industrialization which may significantly contribute to the greenhouse gas emissions and rising global temperatures^2^. It is essentially important to take necessary steps to minimize the heat generated by landfills in order to reduce its impact on the environment. The Paris Agreement, signed by nations in 2015, aims at limiting the global warming to below 2 °C and pursuing the efforts to limit it to 1.5 °C above pre-industrial levels^3,4^. This can be attained by promoting recycling and composting strategies, adopting clean energy sources, and implementing efficient waste management practices in landfills^5,6^. Reducing the heat generated by landfills is one of the many measures that must be taken to achieve the goals set out in the Paris Agreement and prevent the worst impacts of climate change.

The growing energy consumption and utilization of environmentally harmful methods in landfills have become a cause for alarm, eventually requiring necessary actions for environmental preservation. The landfill leachate can result in serious damage to landfill infrastructure and affect the surrounding residential community and biosphere^7^. Leachate management and recirculation in landfills can help lower both heat and leachate generation rate^8^. By recirculating leachate back into the landfill, the organic matter in the waste is further decomposed which reduces the amount of organic material available for heat generation^9,10^. Moreover, mitigation of leachate helps to cool down the waste material, reducing the amount of heat generated within the landfill^11,12^. In addition, reintroduction of leachate in landfills results in improved aeration within the landfill, promoting the growth of aerobic bacteria and reducing the accumulation of heat and leachate^13,14^. This is eventually helpful in preventing its leakage to the nearby water sources, and hence, any water contamination. Beside reducing the environmental risk, the leachate management also lowers the amount of leachate by proper treatment and disposition. Therefore, the practice of leachate management and recirculation in landfills is an effective measure to reduce the heat generated by landfills and lower the amount of leachate generation. These practices help to improve the sustainability and efficiency of waste-to-energy power plants and promote environmental protection.

Investors are taking notice and recognizing the financial potential of leachate management, as they see it as a promising revenue source^15^. By 2027, the waste management industry is projected to generate substantial profits and become a $229 billion industry^16^. Developed countries are actively promoting waste-to-energy processes as they see the financial benefits, while some countries view garbage as a threat and others as an opportunity^17^. To take advantage of the carbon credits offered by the government, many cities are investing in proper leachate management systems, resulting in tax subsidies and lower electricity costs^18^. On the other hand, failure to properly manage and treat leachate can unleash a host of devastating consequences on the environment^19^. This toxic mixture of liquids, chemicals, and pollutants can seep into the groundwater, contaminating drinking water sources and putting public health at risk^20^. For instance, it has been found that the soil and groundwater near a landfill site in Chittagong, Bangladesh were contaminated with high levels of heavy metals due to leachate seepage, eventually becoming unsafe for human consumption and irrigation^21^. Uncontrolled leachate can also poison the soil, choking the growth of vegetation and threatening the wildlife. The release of noxious gases and odors from landfills can also lead to air pollution, affecting the air quality and posing a serious health hazard for nearby communities. A study reported that leachate spill from the Bhalswa landfill in New Delhi, India, led to the release of methane gas and unpleasant odors, contaminated a nearby canal and posed a risk to aquatic life and water quality^22^. Moreover, the accumulation of leachate increases the risk of fires within the landfill, releasing toxic gases and further contaminating the environment. Additionally, the presence of leachate can provide a breeding ground for pests and rodents, leading to the spread of diseases. These recurring events necessitate a full-scale leachate management system. The repeated instances of harm caused by unmanaged leachate emphasize the significance of implementing a heat transfer system through leachate recirculation. This approach not only helps maintain the internal temperature of landfills, but also maximizes methane generation and minimizes carbon dioxide content. Despite its potential, the landfill heat generation has not been explored much for its rich thermos-chemical energy. However, the increasing production of landfill leachate offers an opportunity to harness its heat-capturing capabilities and utilize it as a source of electricity for local communities. This will not only help to reduce the negative impacts of landfills on the environment but will also provide a sustainable solution for energy generation.

An organic rankine cycle (ORC) system can effectively harness this waste heat energy for electricity production^23^. However, widespread implementation of this integrated landfill leachate-ORC system is hindered by poor leachate management practices. The global adoption of such an integrated landfill leachate-ORC system is restricted due to lower efficiency of the combined system which is primarily owing to ineffective leachate utilisation techniques. Pilot studies have investigated the impacts of various parameters on leachate extraction^24,25^, but the optimization of operating conditions for optimal heat extraction and methane generation remains seldomly explored. There is a lack of comprehensive analysis on the physio-chemical characteristics of leachate and their effects on the systems’ performance in previous studies. In the present study, a system has been developed near a landfill to harness the heat generated by leachate fluid. The hot leachate is pumped from various depths of the landfill into a heat exchanger, which is part of an ORC system in the cogeneration cycle. The heat exchanger converts the thermal energy into mechanical power, which is then used to generate electricity for the nearby community. A part of the energy produced by the system is used to power the pumps that extract the leachate from the landfill and circulate it back into the waste, where it acts as a source of moisture and nutrients. This innovative system provides a sustainable solution for managing uncontrolled leachate and high temperatures in landfills, and effectively combines thermal energy extraction with energy supplementation.

Since the performance of landfills is influenced by different physical and chemical parameters, it is important to identify these parameters and experimentally examine their impacts on the overall performance of the landfills. In previous studies, researchers have employed various MCDM methods for landfill optimization. Shahnazari et al.^26^ used combined analytical hierarchy process-technique for order performance by similarity to ideal solution (AHP-TOPSIS) method to study thermal methods in the waste-to-energy sector. Abdolkhaninezhad et al.^27^ used Fuzzy-ANP and Fuzzy-TOPSIS with bow ties method for risk assessment and management in leachate circulation process for municipal landfills so as to identify various significantly important environmental and health-safety risks sources. Zhang et al.^28^ evaluated membrane concentrate leachate circulation and treatment technologies, simultaneously ranking them based on treatment efficiency, operational cost, environmental benefit, and process stability using AHP. Özdemir et al.^29^ evaluated four different options for Municipal Solid Waste (MSW) landfill leachate treatment using Multi-criteria Decision-making (MCDM) techniques based on technical, economic and environmental aspects and the most appropriate option was selected using ANP and PROMETHEE methods. The present study mainly focusses on exploring favourable conditions for leachate recirculation in landfills in order to address the waste management issue by improving the leachate management operations.

There might be prior studies that has explored the impact of varying physicochemical properties in landfills to maximize the methane and simultaneously minimize carbon dioxide through leachate recirculation^30^. However, the literature reveals limited number of articles focusing on the sustainable performance of landfills integrated with leachate recirculation processes powered by a separate ORC system, so as to increase the profitability of the plant. Previous studies conducted in this domain lack the application of systematic approach to optimize multiple performance characteristics to achieve sustainability and profitability for waste to energy plant by leachate recirculation. This work presents a comprehensive investigation of the impact of leachate recirculation on methane generation and landfill dynamics. It addresses the complex interplay between leachate flow rates, hydraulic conductivity, and internal temperature. It also determines the optimal conditions required for efficient methane production. Moreover, use of an appropriate design of experiments for experimental investigation along with the application of MCDM and statistical methods to optimize the physical and chemical properties for combined methane production and electricity generation through leachate recirculation is a new concept and rarely explored in previous literatures. This study presents a novel approach to find landfill input parameters which may enhance the profitability and sustainability of the waste-to-energy power plants. An experimental investigation was conducted to study the effects of varying physical and chemical properties in landfills for effective leachate management. The optimal input parameters for achieving the best overall performance characteristics are derived.

The experiments were carried out based on Taguchi’s orthogonal experimental array design. The experimental values of the output parameters were used to find an overall performance score using integrated MCDM methods, where ‘Method based on the removal effects of criteria (MEREC)’ was used to find importance weights of the output parameters and ‘Proximity indexed value (PIV)’ method was deployed for finding the performance scores of the landfill operation from the values of the output parameters. The signal-to-noise ratios of the performance scores were subsequently analyzed using statistical techniques, i.e. analysis of mean (ANOM) and analysis of variance (ANOVA) to determine best combination of the input parameters and to establish their significance on the overall performance. Henceforth, the objectives of this study are to:

- Investigate the impact of input waste characteristics, on the outcomes such as CO_2_ emission, methane emission, heat generation, and temperature of the trench during the landfill operations.
- Identify the optimal combination of input parameters to achieve optimum performance measures in landfill operations, with specific values for waste particle size, waste addition, inorganic content in waste, leachate recirculation rate, and landfill age.
- Assess the relative significance of different input parameters on the multiple performance measures of landfill operations, ranking them in order of their influence.

The present work offers various contributions to the research in comparison to the previous research works. Unlike prior research, this study considers multiple factors, including leachate flow rates, hydraulic conductivity, and internal temperature, to provide a holistic understanding and more generalized findings. It identifies the optimum temperature range for maximum methane production and emphasizes the importance of managing landfill temperature within this range. The study addresses potential challenges such as water collection system clogging and leachate solidification, offering practical solutions to enhance leachate recirculation practices. Moreover, it proposes the implementation of a sophisticated monitoring and control facility to ensure optimal landfill conditions and methane generation. These novel aspects differentiate this study from previous research and provide valuable insights for sustainable landfill management and improved methane recovery.

## Leachate management: process and experimental setup

Despite the pilot studies that examined the influence of various parameters on the extraction systems in landfills, the impact of leachate recirculation on heat extraction and methane generation for optimizing operating parameters remains a crucial area for energy production^31^. Consequently, to ensure sustainable management of leachate, identification of critical input parameters and their range, selection of important landfill performance measures (PMs) and optimization of input parameter have been done in the present study. Four main PMs of the waste-energy landfill viz. leachate recirculation, methane production, CO_2_ production, and heat mitigation have been considered in the contemporary investigation. Subsequent sections describe the collection techniques and parameters considered in the present study.

### Site location

Introducing an innovative energy solution, the present setup comprises of an integrated system that harnesses the power of the hot leachate generated from landfills to produce electricity for neighbouring groups. Located in rich population centres, these landfills are strategically built to efficiently collect a large quantity of leachate while reducing treatment costs. Furthermore, the close proximity of these landfills to communities provides easy accessibility to electricity. In addition to utilizing methane, these landfills can also employ other alternative sources of energy readily available on site, such as geothermal and solar energy^32^. With a focus on circular economy and emissions-free operations, these leachate management programs are making a positive impact on the environment.

The experimental data for the present study was collected from a landfill located in Okhla, Jamia Nagar, New Delhi, India. This landfill utilizes a well-established management system to closely monitor and manage the leachate since 2014. The focus of the study is on organic waste produced by the densely populated local communities nearby the landfill. The landfill experiences a wide range of temperatures, with colder temperatures of 2–10 ℃ during the winter and warmer temperatures of 35–50 ℃ during the summer. The average annual precipitation in the area is 617 mm. In order to gather comprehensive data, a total of 25 different trenches were analysed, with the average values serving as the basis for the study’s findings.

**Figure 1:**
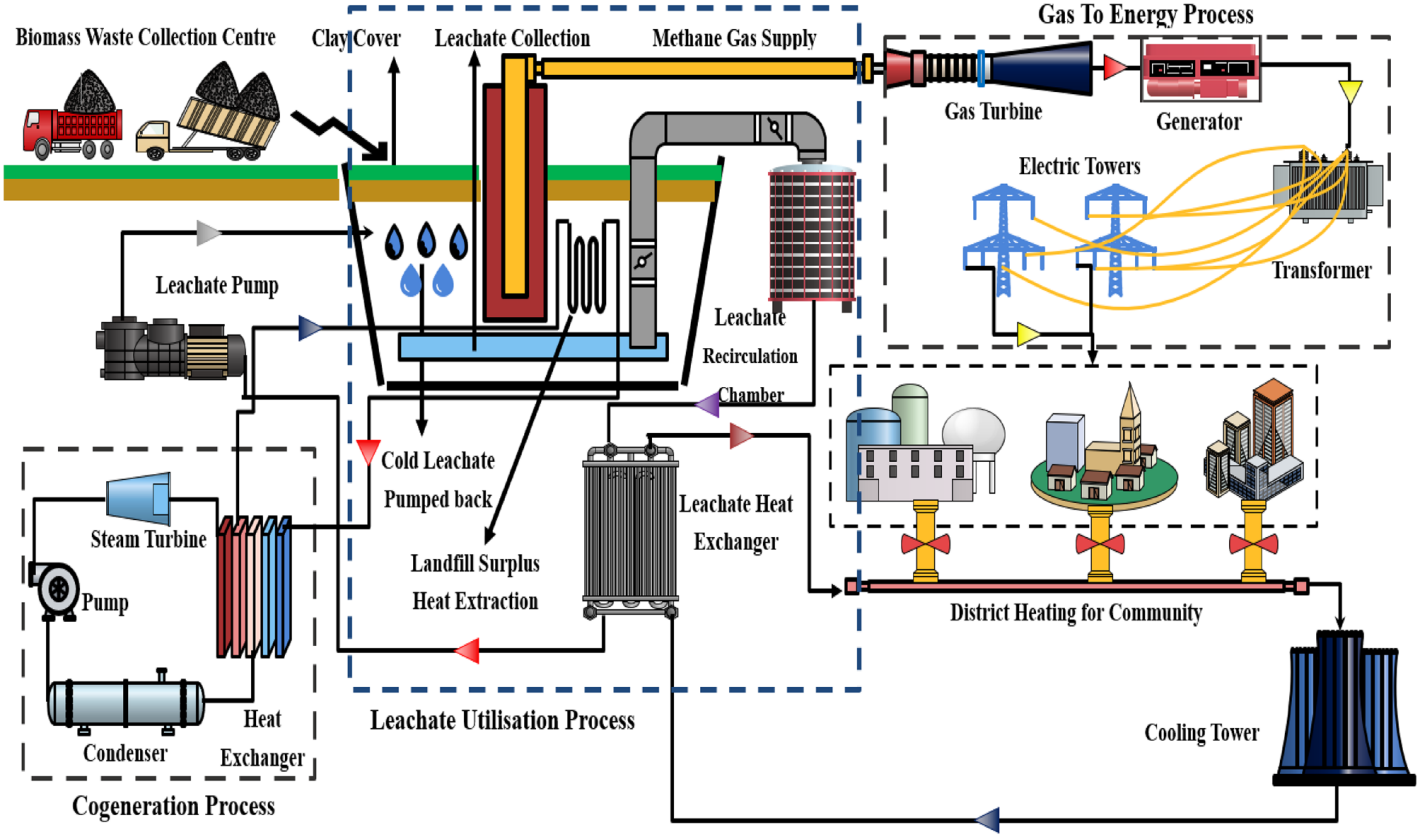
Schematic arrangement of leachate recirculation process in waste to energy plant.

The process of landfill leachate recirculation and waste heat utilisation is depicted in the schematic shown in Fig. 1.

Initially the process begins with dumping of the agricultural waste products in fine shredded form into the trench forming the first layer. Several layers are further added in subsequent days to completely fill the trench. The trench is pre-equipped with leachate collection system, installed at the bottom of the trench where surplus leachate is collected and pumped out of the system. This process also helps to minimize the release of pollutants into the environment by preventing the contaminated water from reaching the groundwater and surface water. Moreover, a heat capturing arrangement has been installed at the left portion of the trench to capture the thermal energy available due to decomposition of waste. The landfill gas generated from the decomposition of waste typically consists of methane and carbon dioxide, both of which are potent greenhouse gases. The methane produced in the trench is collected and diverted to gas turbine for electricity production which is then distributed to a nearby residential community by suitable electrical equipment. By capturing the landfill gas and using it to generate energy, the emissions of these gases can be reduced, thereby mitigating the environmental impact of the landfill^33^. On the other hand, leachate collected from the trench is diverted into the leachate collection chamber. The hot leachate is then transported into the heat exchanger where the thermal energy is exchanged with the cold stored water available in the cooling tower. The heat collected in the water is utilised in residential houses for district heating while the cold leachate is redirected towards the centrifugal pump which sucks the leachate back into the landfill thereby maintaining the inside temperature of landfill below 30 ℃ which is favourable for methane production. The treated leachate is returned to the landfill to increase the moisture content of the waste, which enhances the biodegradation of organic matter and ultimately reduces the amount of waste that needs to be landfilled. Furthermore, simultaneously heat is being extracted through the trench which is supplied to a cogeneration system which produces power in the process. One of the major advantages of landfill leachate recirculation is the generation of heat from the microbial activity in the landfill. The heat generated from the decomposition of organic matter can be harnessed to produce energy, such as electricity or heat, which can be supplied to the nearby cogeneration system. This surplus power is utilised by the centrifugal pump of the leachate recirculation system, eventually increasing the sustainability aspect of the process. In addition, the energy produced from landfill gas-to-energy projects provides a sustainable source of energy, which reduces the reliance on non-renewable fossil fuels. In addition, the energy produced from landfill gas-to-energy projects provides a sustainable source of energy, which reduces the reliance on non-renewable fossil fuels. The Leachate Pollution Index (LPI) is a singular numeric indicator assessing the potential toxicity of leachate in a specific landfill, usually on a scale from 5 to 100, determined using the Delphi technique. In the case of the Okhla landfill, the LPI exhibits seasonal variation, ranging between 28 and 32. These fluctuations in LPI highlight the urgent need for immediate attention to address leachate concerns through recirculation or treatment processes. This action is crucial to safeguard groundwater quality, as leachate infiltration poses a risk of contaminating and degrading the groundwater.

The moisture content inside the landfill bioreactor, after the landfill is closed, can be obtained using equation given below:

1 $${\theta _{{cc}}}  = 0.6 - 0.5\left( {\frac{w}{{4536^{{ + w}} }}} \right),$$

where ${\theta }_{cc}$= Moisture content within the landfill ($\mathrm{g}/{\mathrm{m}}^{3}$), $w$ = Specific humidity of the waste ($\mathrm{g}/\mathrm{kg}$).

### BIOLEACH model for leachate management

**Figure 2:**
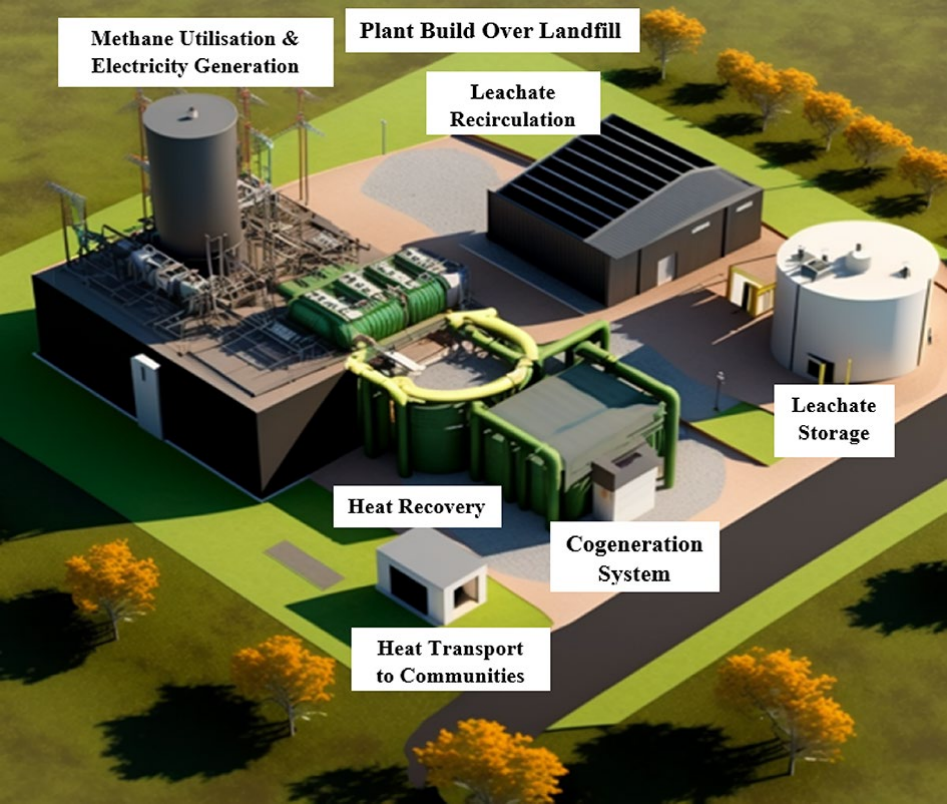
3-D model developed from BIOLEACH-LANDGEM software.

The use of bioreactor landfill models offers a unique solution to evaluate both leachate and biogas production simultaneously. With its innovative approach, the model takes into consideration the actual chemical composition of the waste, thereby simulating actual conditions of the landfill more accurately. Further, the bioreactor management strategy allows the calculation of optimal leachate recirculation amount with the aim of maximizing biogas production and maintaining the appropriate water content within the landfill. BIOLEACH-LANDGEM is a cutting-edge mathematical model that supports real-time decision making for urban solid waste landfills as shown in Fig. 2.

By modelling the production of both leachate and biogas in a synergistic manner, the model can simulate the behaviour of the landfill as a bioreactor and the effect of recirculating leachate stored within it. This makes BIOLEACH a valuable tool for optimizing the management of landfills and promoting sustainability.

It is a well-known fact that temperatures above 55 ℃ can hinder gas production, making it essential to remove and harness the excess heat. The hot and humid climates of summer in New Delhi can cause internal temperatures in the landfill to soar up to 80–85 ℃, making leachate management and recirculation a top-notch priority. The data for this study was gathered over a 6-month period from January to June, using multiple extraction techniques, including vertical and horizontal systems. A pumping system was installed at various depths in the vertical direction to remove hot leachate from the system, while cold leachate was sprayed back into the landfill through the horizontal system, covering a greater volume of organic waste. The vertical pumping setup was installed during the waste accumulation phase, with the horizontal spray system added in subsequent layers after each compaction phase. The leachate recirculation rate during the extraction phase was maintained at a velocity of 0.3 m/s, while the spraying flow rate was 0.25 m/s. Any surplus leachate was stored depending on the season. The internal thermal energy within the landfill was monitored at regular intervals using temperature sensors placed at specific locations.

## Methodology

### Landfill multiple performance optimization process

**Figure 3:**
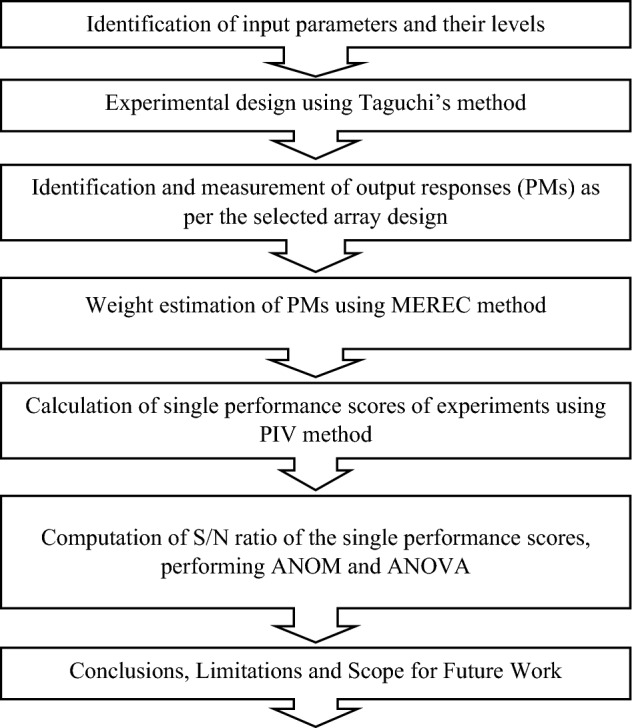
Procedure for multi-performance optimization of landfill.

The process of optimizing the input parameters of landfill operations involves several important steps, as depicted in Fig. 3. The main steps include identification of input variables and their levels, identification of PMs, selection of appropriate combinations of input parameters and their levels for testing, application of MCDM for determination of single performance score, computation of S/N ratio of single performance, use of ANOM and ANOVA for finding the optimal setting of input parameters and their significance.

The detailed description of the steps of the algorithm shown in Fig. 3 are given in the following section.

### Selection of input parameters

The selection of input parameters is a crucial aspect of any experimental investigation, as it greatly impacts the outcomes. In traditional approaches, the parameters are often chosen based on the machines or techniques available at the landfill site. However, in the present analysis, a more informed approach has been taken. The input variables were selected based on previous studies and their known impact on the specific outcomes being considered. Five key input parameters namely waste particle size (WPS), waste addition (WA), inorganic content in waste (IO), leachate recirculation rate (LRR) and landfill age (LA) were selected in this study. The selection of these parameters was based on a comprehensive literature survey that established their relationship with the outcomes and their feasibility for the study. To determine the distinct ranges of the input parameters, the feasible variety that greatly impacts the outcomes was recognized. The selected ranges of input parameters are as follows: WPS lies between 6 and 18 ppm, WA varies from 20 to 100 Ktoe, IO is within the range from 2 to 10%, LRR falls in between 100 and 300 L/day, and LA ranges from 1 to 5 years. The input parameters and their levels are shown in Table 1.

**Table 1:** The input parameters and their levels.

Symbol	Input parameter	Unit	Level-1	Level-2	Level-3	Level-4	Level-5
*A*	Waste particle size	Ppm	6	9	12	15	18
*B*	Waste addition	Ktoe	20	40	60	80	100
*C*	Inorganic content in waste	%	2	4	6	8	10
*D*	Leachate recirculation rate	l/day	100	150	200	250	300
*E*	Landfill age	Years	1	2	3	4	5

The focus of this study was based on leachate utilisation within the landfills, and for this reason, atmospheric constraints were not taken into consideration. Despite the fact that wind temperatures and earth temperature might cause minor variations in the leachate generation rate, however, they were not considered to be significant forces in this study. This is because when the landfill is covered, the climatic variations no longer have a dominant impact on the leachate generation rate. Eventually, they were not considered in this study, thereby mainly focusing on the leachate mitigation within the landfill after the covering process was completed. By excluding these atmospheric constraints, the study can delve deeper into the inside conditions of landfills affecting leachate generation within the landfill itself. One of the limitations of the present study is that certain critical variables such as leachate quality, moisture content, temperature, microbial activity and quantum of waste that are crucial for a comprehensive understanding of heat development in the landfill, have been treated as constant for all experiments rather than being included as dynamic parameters. Due to its proximity to one of the largest vegetable markets, Okhla landfill receives a significant influx of waste vegetables and fruits annually. For the purpose of this analysis, only food-based waste, such as vegetables and fruits, was considered. This waste stream contained a small and variable quantity of inorganic matter, primarily paper, with organic matter ranging from 90 to 98% and inorganic matter ranging from 2 to 10%. Given the predominant organic composition, a uniform degradation rate of 0.035 per year was assumed for all experimental test runs. The waste was estimated to have a moisture content of approximately 60%. The analysis was conducted during the pre-monsoon period, and the Initial Leachate Quality was characterized by a pH of 6.5, COD of 60,000 ± 5.01 mg/L, BOD of 35,000 ± 4.14 mg/L, TDS of 50,000 ± 11.22 mg/L, and TCB of 350 ± 40.1.

### Taguchi’s design of experiment

Design of experiments is required to collect values of the PMs of a process or system for different setting of the input parameters. In full factorial design, the experiments are conducted at all possible combination of factors and their levels. However, when the number of factors or their levels to be studied are large, the process of experimentation according to full factorial design turn out to be very costly and time consuming. Moreover, it becomes unmanageable to control the undesired effects caused by material heterogeneity (due to large quantity of material for more runs), or surrounding conditions, etc. To overcome this problem, fractional factorial design has been developed wherein experiments are required to be conducted only for a limited combination of factors and their levels. However, the design of experiments using fractional factorial design requires good statistical knowledge on the part of the experimenter, thereby limiting the applicability and ease of conducting experiments in practice. A simpler approach of design of experiment was later proposed by Genichi Taguchi, who suggested the use of Orthogonal Arrays (OAs) for systematically finding the minimum set of factor-and-level combinations to be tested in an experiment, thereby reducing the time and cost of experimentation. The Taguchi’s design is based on the following two concepts:

(i) Design the product so that all products perform as identically as possible; and,
(ii) Design the product to perform in the best manner most of the time.

The Taguchi’s approach employs a statistical measure known as the signal-to-noise (S/N) ratio, to evaluate the performance of the design parameters. This ratio allows for a quantitative assessment of both the location and dispersion of the measured responses and helps to identify the best levels of the input parameters in order to achieve the highest S/N ratio. By using the S/N ratio, the raw data is transformed into meaningful information that can be used to determine the most robust design. The end result is a design that is less sensitive to noise factors and more likely to perform consistently over time. This makes the S/N ratio an effective tool for identifying the optimal levels of the design parameters, which can lead to improved outcomes and greater efficiency in the design and operation of the system which in this case is landfills.

In the present work Taguchi’s experimental design was employed to reduce the number of experiments to be conducted and an L_25_ orthogonal array was selected for which the experimental layout is shown in Table 2.

**Table 2:** The experimental layout of Taguchi’s L_25_ orthogonal array design.

Experiment no.	Waste particle size (A)	Waste addition (B)	Inorganic content in waste (C)	Leachate recirculation rate (D)	Landfill age (E)
1	6	20	2	100	1
2	6	40	4	150	2
3	6	60	6	200	3
4	6	80	8	250	4
5	6	100	10	300	5
6	9	20	4	200	4
7	9	40	6	250	5
8	9	60	8	300	1
9	9	80	10	100	2
10	9	100	2	150	3
11	12	20	6	300	2
12	12	40	8	100	3
13	12	60	10	150	4
14	12	80	2	200	5
15	12	100	4	250	1
16	15	20	8	150	5
17	15	40	10	200	1
18	15	60	2	250	2
19	15	80	4	300	3
20	15	100	6	100	4
21	18	20	10	250	3
22	18	40	2	300	4
23	18	60	4	100	5
24	18	80	6	150	1
25	18	100	8	200	2

Since S/N analysis can be performed only on single performance score, it is pertinent to combine the multi-performance results of landfill operations using MCDM technique so that single performance scores can be obtained corresponding to each experimental run. An integrated MCDM technique, MEREC-PIV was implemented for obtaining the single performance scores. The MEREC method was used to determine the objective weights of the PMs, whereas PIV method was used to find the single performance scores of the experimental runs. The single performance scores were subsequently transformed to the corresponding S/N and then analysis of mean (ANOM) and analysis of variance (ANOVA) were performed. ANOM was used to identify the optimal combination of the five input parameters which can yield the optimum multiple performances, whereas ANOVA was conducted to find significance of the input parameters for the multiple performances.

### Performance measures and their examination process

Once the input parameters and their desired ranges have been selected, it is important to understand how they impact the considered PMs. These PMs play a crucial role in shaping the perspectives of landfill owners, who rely on the profits generated from the huge investments. To gain this understanding, the input parameters were incorporated into mathematical formulas that describe the behaviour of the PMs. These formulas were then analysed to determine how changes in the input parameters affect the PMs. To optimize the PMs for optimal input parameters, MCDM technique along with ANOM and ANOVA was used.

#### Thermal energy generation rate

The decomposition of waste results in the production of heat, methane gas, and CO_2_. This chemical reaction is exothermic, meaning that it releases a significant amount of heat, which in turn elevates the internal temperature of the landfill. In order to contemplate the effects of physio-chemical properties of waste, a heat model based on a semi-empirical time dependent logarithmic decay formulation as given in Eq. (2) was used:

2 $$TE=A\left(\frac{t}{B+t}\right)\left(\frac{C}{C+t}\right) {e}^{-\sqrt{\frac{t}{D}} },$$

where $TE$ is thermal energy (W/m^3^), *t* is time period (days), $A$ is zenith thermal energy factor (W/m^3^), $B$ and $C$ are time period constants (days), and $D$ is decay factor (days).

The heat generated in landfills is a crucial aspect which should be studied to understand the conditions inside the landfill. To determine the heat rate, simulations were carried out for different trenches in a landfill located in New Delhi, for a period of approximately 150 days. The simulations considered the temperature dependency, with temperatures ranging between 30 °C and 55 °C. The heat rate is dependent on the organic and methane content within the landfill, with higher inorganic substances inhibiting the anaerobic reaction and limiting the heat development phase.

#### Methane and carbon di-oxide generation

The production of methane gas is a significant performance measure of landfill operations, and it is important to manage it effectively. Methane is a highly flammable gas that can be harmful to the environment if it escapes into the atmosphere, as it is one of the major greenhouse gases. To prevent this, pipes were installed underground to extract the gas through gas wells. The LANDGEM software was used to simulate the power generated from the gas. The model is based on a first-order decay rate that estimates the annual amount of gas produced for a specific landfill. Two variables play a crucial role in calculating methane generation: the yearly filling rate and waste particle size. The analysis was conducted for a period of 1 to 5 years. The model used the potential methane generation capacity (L_0_) and methane generation rate (k), which were 140 m^3^/Mg and 0.1 yr^–1^ respectively. Previous research has shown that methane generation is comparatively higher in landfills that have a leachate recirculation facility. This increase in methane generation was found to be around 15% compared to landfills without recirculation facilities. Additionally, the time required for the waste to reach stability decreased from 33 to 21%.

#### Temperature of landfill

The internal temperature of the landfill is not influenced by the surrounding climate, but the bio-chemical processes within the landfill are crucial. The decomposition of waste releases heat through an exothermic reaction. While higher temperatures are ideal for methane generation, temperatures exceeding 55 ℃ halt the anaerobic reaction, causing a halt in methane production. Hence, it is crucial to continuously monitor the temperature of the waste to optimize methane production and minimize CO_2_ generation. Previous research has found that a temperature range between 25 and 30 ℃ is optimal for methane generation, while temperatures below 10 ℃ hinder the bacterial decomposition of organic waste.

### Weight estimation of performance measures using method based on the removal effects of criteria (MEREC)

The MEREC method was proposed recently in 2021 by Keshavarz-Ghorabaee et al.^34^ to find the objective weight of criteria. The concept of MEREC lies in evaluating the relative importance of criteria by calculating the effect on the performance of the alternatives on removal of each criterion. The criteria producing the higher effect on the alternative’s performance is considered more important and are assigned larger weights. In the present work, the PMs represent the criteria whereas the experimental runs represent the alternatives. The MEREC method involves a simple computational procedure which has attracted wide applications in decision making problems. The steps used for determining criteria weights in MEREC method are as follows:

*Step 1* Construction of the decision matrix, $X$: The decision matrix is formed by arranging the scores of alternatives corresponding to each criterion in 2-D array, as depicted in Eq. (3):

3 $$X={\left[{X}_{ij}\right]}_{m\times n}= \left[\begin{array}{cccccc}{x}_{11}& {x}_{12}& \dots & {x}_{1j}& \dots & {x}_{1n}\\ {x}_{21}& {x}_{22}& \dots & \dots & \dots & {x}_{2n}\\ \dots & \dots & \dots & \dots & \dots & \dots \\ {x}_{i1}& \dots & \dots & {x}_{ij}& \dots & {x}_{in}\\ \dots & \dots & \dots & \dots & \dots & \dots \\ {x}_{m1}& \dots & \dots & {x}_{mj}& \dots & {x}_{mn}\end{array}\right],$$

where $i= 1, 2, \dots , m; j=1, 2, \dots , n$. where ${x}_{ij}$ represents the value of *i*th alternative with respect to *j*th criterion, *m* indicates the number of alternatives, and *n* indicates the number of criteria. In the present work the scores (${x}_{ij}$) represent the values of all the four PMs for all the twenty-five experimental runs.

*Step 2* Normalization of decision values: The decision values (${x}_{ij}$) of Eq. (3) are linearly normalized by transforming all the criteria, beneficial or non-beneficial, into the minimization type criteria, using Eq. (4):

4 $$ r_{{ij}}  = \left\{ {\begin{array}{*{20}c}    {\frac{{\mathop {\min }\limits_{k} \left( {x_{{kj}} } \right)}}{{x_{{ij}} }}} & {if \; j \in {\text{Beneficial}} \; {\text{criterion}}}  \\    {\frac{{x_{{ij}} }}{{\mathop {\max }\limits_{k} \left( {x_{{kj}} } \right)}}} & {if \; j \in {\text{Non-Beneficial}} \; {\text{criterion}}}  \\   \end{array} } \right. $$

*Step 3* Calculation of alternatives’ performances: The performance of alternative is calculated using a logarithmic function, as expressed in Eq. (5), such that alternatives with smaller normalized values yield better performance and visa-versa.

5 $${P}_{i}=\mathrm{ln}\left[1+\left(\frac{1}{n}\sum_{j}\left|\mathrm{ln}\left({r}_{ij}\right)\right|\right)\right].$$

*Step 4* Calculation of alternatives’ performances on removal of each criterion: The performance is calculated again, however, by removing one criterion at a time. This can be mathematically expressed as given in Eq. (6):

6 $${P}_{ij}{\prime}=\mathrm{ln}\left[1+\left(\frac{1}{n}\sum_{k, k\ne j}\left|\mathrm{ln}\left({r}_{ij}\right)\right|\right)\right].$$

The alteration in the performance can further be used to identify the contribution of each criterion in the overall performance of an alternative.

*Step 5* Evaluation of removal effect: In this step, the alteration in the performance of ith alternative is evaluated which indicates the contribution of each criterion in the overall performance of that alternative. The removal effect is calculated as given by Eq. (7) by summing the absolute deviations of the performance obtained by removing the criterion from the actual performance obtained when all the criteria were considered.

7 $${E}_{j}=\sum_{i}\left|{P}_{ij}{\prime}-{P}_{i}\right|.$$

*Step 6* Calculation of objective weights of criteria: The criteria weights are determined from the removal effects using Eq. (8):

8 $${w}_{j}= \frac{{E}_{j}}{\sum_{j}{E}_{j}}.$$

Compared to other commonly used objective weight estimation techniques, like CRITIC, Entropy or standard deviation methods, the MEREC method produces weights with high variance, thereby distinguishing more efficiently between different criteria. Moreover, the weights obtained using MEREC method are relatively more stable^34^. Hence, the present work employs this method to estimate the weights of the different performance measures.

### Proximity indexed value (PIV) method for single performance scores calculation 

The PIV method was developed by Mufazzal and Muzakkir in (2018)^35^, to minimize rank reversal problems in decision making. The rank reversal is a very common phenomenon in decision making, wherein the ranks of the alternatives get reversed on addition or removal of alternative/s from the list of alternatives that are to be evaluated. The PIV method identifies only the ideal best alternative and computes the dispersion of other alternatives from this ideal reference. Owing to its simple computational steps yet with robust results against rank reversal, this method has been widely accepted in literature, and therefore, has been used in the present work.

The PIV method involves the following main steps of computation:

*Step 1* Construction of decision matrix as given by Eq. (3).

*Step 2* Data normalization using Eq. (9), as follows:

9 $${n}_{ij}=\frac{{x}_{ij}}{\sqrt{\sum_{i=1}^{m}{x}_{ij}^{2}}},$$

where ${n}_{ij}$ is the normalized value of ${x}_{ij}$.

*Step 3* Determination of the weighted normalized values (${v}_{ij}$) using Eq. (10):

10 $${v}_{ij}={w}_{j}{x}_{ij,}$$

where ${w}_{j}$ represents the weight of the criterion *j* and ${v}_{ij}$ indicates the weighted normalized value for *i*th alternative with respect to *j*th criterion*.*

*Step 4* Evaluation of weighted proximity indices ($${u}_{ij})$$: The weighted proximity index indicates the deviation of each alternative from the ideal best, and it is calculated according to the beneficial or non-beneficial attribute of the criteria as given by Eq. (11):

11 $$ u_{{ij}}  = {\text{ }}\left\{ {\begin{array}{*{20}l}    {v_{{\max }}  - v_{{ij}} } \hfill & {{\text{if}}\;j \in {\text{Beneficial criterion}}} \hfill  \\    {v_{{ij}}  - v_{{\min }} } \hfill & {{\text{if}}\;j \in {\text{Non - Beneficial criterion}}} \hfill  \\   \end{array} } \right.,$$

where $${v}_{\mathrm{max}}=\underset{i}{\mathrm{max}}({v}_{ij})$$ and $${v}_{\mathrm{min}}=\underset{i}{\mathrm{min}}({v}_{ij})$$.

*Step 5* Calculation of overall proximity index (${d}_{i}$) and ranking of alternatives:

The weighted proximity index is aggregated to find the overall proximity of an alternative with the ideal best, and it is calculated using Eq. (12) as follows:

12 $${d}_{i}={\sum }_{j=1}^{n}{u}_{ij}.$$

The alternatives having smaller values of ${d}_{i}$ indicate lesser deviation from the ideal best. Hence, the alternatives are ranked in increasing order of ${d}_{i}$ values.

## Results and discussion 

### Determination of criteria (performance measures) weights using MEREC method

The experimental values of the PMs were obtained for all the twenty-five experimental settings. These values were used to construct the decision matrix, as shown in Table 3.

**Table 3:** Decision matrix showing the experimental results for the performance measures of landfill operation.

Experiment no.	CO_2_ emission (%)	Heat generation (kW)	Methane emission (%)	Temperature of the trench (℃)
1	20	6.1	39	41
2	18	5.9	40	38
3	15	6.3	43	36
4	14	6.4	44	35
5	19	6	42	38
6	18	6.2	41	37
7	21	6.3	42	39
8	23	6.5	45	40
9	18	6.8	43	39
10	13	7.2	48	36
11	17	6.1	42	35
12	21	5.9	39	44
13	19	6.7	43	41
14	20	7.3	47	36
15	21	6.7	47	35
16	25	5.5	37	43
17	24	5.9	35	41
18	16	7.4	49	33
19	18	7	45	35
20	22	6.4	42	40
21	27	5.4	33	38
22	24	6.2	39	36
23	21	6.5	42	44
24	20	6.4	40	42
25	24	6.3	38	40

Among the four response variables, the ‘CO_2_ emission’ and ‘temperature of the trench’ are non-beneficial PMs, whereas the other two, namely, ‘heat generation’ and ‘methane emission’ are beneficial PMs. Therefore, the decision values of Table 3 were normalized according to Eq. (4). The normalized decision matrix is shown in Table 4.

**Table 4:** Normalized decision matrix.

Experiment no.	CO_2_ emission (%)	Heat generation (kW)	Methane emission (%)	Temperature of the trench (℃)
1	0.7407	0.8852	0.8462	0.9318
2	0.6667	0.9153	0.8250	0.8636
3	0.5556	0.8571	0.7674	0.8182
4	0.5185	0.8438	0.7500	0.7955
5	0.7037	0.9000	0.7857	0.8636
6	0.6667	0.8710	0.8049	0.8409
7	0.7778	0.8571	0.7857	0.8864
8	0.8519	0.8308	0.7333	0.9091
9	0.6667	0.7941	0.7674	0.8864
10	0.4815	0.7500	0.6875	0.8182
11	0.6296	0.8852	0.7857	0.7955
12	0.7778	0.9153	0.8462	1.0000
13	0.7037	0.8060	0.7674	0.9318
14	0.7407	0.7397	0.7021	0.8182
15	0.7778	0.8060	0.7021	0.7955
16	0.9259	0.9818	0.8919	0.9773
17	0.8889	0.9153	0.9429	0.9318
18	0.5926	0.7297	0.6735	0.7500
19	0.6667	0.7714	0.7333	0.7955
20	0.8148	0.8438	0.7857	0.9091
21	1.0000	1.0000	1.0000	0.8636
22	0.8889	0.8710	0.8462	0.8182
23	0.7778	0.8308	0.7857	1.0000
24	0.7407	0.8438	0.8250	0.9545
25	0.8889	0.8571	0.8684	0.9091

The overall performances of the experimental runs before and after removing the criteria were calculated using Eq. (5), and their values are listed in the second column of Table 5.

**Table 5:** Values of overall performance before and after removing criteria.

Experiment no.	${S}_{i}$	${S}_{i1}^{\mathrm{^{\prime}}}$	${S}_{i2}^{\mathrm{^{\prime}}}$	${S}_{i3}^{\mathrm{^{\prime}}}$	${S}_{i4}^{\mathrm{^{\prime}}}$
1	0.1526	0.0861	0.1261	0.1161	0.1374
2	0.1892	0.1015	0.1707	0.1486	0.1584
3	0.2638	0.1440	0.2337	0.2116	0.2245
4	0.2895	0.1584	0.2572	0.2342	0.2458
5	0.1916	0.1163	0.1696	0.1405	0.1608
6	0.2098	0.1241	0.1814	0.1649	0.1741
7	0.1755	0.1213	0.1426	0.1236	0.1498
8	0.1721	0.1378	0.1323	0.1046	0.1518
9	0.2274	0.1432	0.1804	0.1732	0.2031
10	0.3354	0.1954	0.2826	0.2661	0.2989
11	0.2340	0.1380	0.2096	0.1851	0.1877
12	0.1193	0.0619	0.0995	0.0815	0.1193
13	0.2034	0.1291	0.1584	0.1479	0.1889
14	0.2538	0.1939	0.1936	0.1828	0.2141
15	0.2330	0.1819	0.1893	0.1604	0.1866
16	0.0565	0.0382	0.0522	0.0291	0.0511
17	0.0806	0.0531	0.0600	0.0669	0.0642
18	0.3223	0.2228	0.2636	0.2480	0.2688
19	0.2631	0.1820	0.2120	0.2017	0.2182
20	0.1636	0.1192	0.1269	0.1111	0.1432
21	0.0360	0.0360	0.0360	0.0360	0.0000
22	0.1449	0.1191	0.1146	0.1081	0.1005
23	0.1566	0.1013	0.1161	0.1036	0.1566
24	0.1632	0.0973	0.1264	0.1214	0.1532
25	0.1196	0.0932	0.0848	0.0878	0.0983

Next, the performance of each experiment was calculated using Eq. (6) by removing each factor, and their values are listed in column 3 to column 6 of Table 5.

The removal effect of each response factor was obtained by calculating the deviation using Eq. (7). The results obtained along with the criteria weight (obtained using Eq. (8)) are provided in Table 6.

**Table 6:** Removal effect of each response factor and their weights.

Experiment no.	CO_2_ emission (%)	Heat generation (kW)	Methane emission (%)	Temperature of the trench (℃)
1	− 0.0666	− 0.0265	− 0.0365	− 0.0153
2	− 0.0876	− 0.0185	− 0.0406	− 0.0308
3	− 0.1198	− 0.0300	− 0.0522	− 0.0393
4	− 0.1312	− 0.0323	− 0.0553	− 0.0438
5	− 0.0753	− 0.0220	− 0.0511	− 0.0307
6	− 0.0858	− 0.0284	− 0.0450	− 0.0357
7	− 0.0542	− 0.0329	− 0.0519	− 0.0256
8	− 0.0343	− 0.0398	− 0.0675	− 0.0203
9	− 0.0842	− 0.0470	− 0.0542	− 0.0243
10	− 0.1400	− 0.0528	− 0.0693	− 0.0365
11	− 0.0960	− 0.0244	− 0.0489	− 0.0463
12	− 0.0574	− 0.0198	− 0.0378	0.0000
13	− 0.0744	− 0.0450	− 0.0555	− 0.0145
14	− 0.0600	− 0.0603	− 0.0711	− 0.0397
15	− 0.0511	− 0.0437	− 0.0726	− 0.0464
16	− 0.0183	− 0.0043	− 0.0274	− 0.0054
17	− 0.0275	− 0.0206	− 0.0137	− 0.0164
18	− 0.0996	− 0.0588	− 0.0743	− 0.0535
19	− 0.0811	− 0.0512	− 0.0614	− 0.0450
20	− 0.0444	− 0.0367	− 0.0525	− 0.0204
21	0.0000	0.0000	0.0000	− 0.0360
22	− 0.0258	− 0.0303	− 0.0368	− 0.0444
23	− 0.0552	− 0.0404	− 0.0529	0.0000
24	− 0.0659	− 0.0367	− 0.0417	− 0.0099
25	− 0.0265	− 0.0348	− 0.0318	− 0.0214
${E}_{k}$	1.6620	0.8373	1.2020	0.7017
${w}_{j}$	0.3775	0.1902	0.2730	0.1594

### Determination of the single performance scores using PIV method

The decision matrix given in Table 3 was normalized as per the relationships given in Eq. (9). The normalized decision matrix is shown in Table 7.

**Table 7:** Normalized decision matrix for PIV method.

Experiment no.	CO_2_ emission (%)	Heat generation (kW)	Methane emission (%)	Temperature of the trench (℃)
1	0.8962	0.4832	1.2064	1.3219
2	0.8066	0.4673	1.2374	1.2252
3	0.6722	0.4990	1.3302	1.1607
4	0.6274	0.5069	1.3611	1.1284
5	0.8514	0.4752	1.2992	1.2252
6	0.8066	0.4911	1.2683	1.1929
7	0.9410	0.4990	1.2992	1.2574
8	1.0307	0.5148	1.3920	1.2897
9	0.8066	0.5386	1.3302	1.2574
10	0.5825	0.5703	1.4849	1.1607
11	0.7618	0.4832	1.2992	1.1284
12	0.9410	0.4673	1.2064	1.4186
13	0.8514	0.5307	1.3302	1.3219
14	0.8962	0.5782	1.4539	1.1607
15	0.9410	0.5307	1.4539	1.1284
16	1.1203	0.4356	1.1446	1.3864
17	1.0755	0.4673	1.0827	1.3219
18	0.7170	0.5861	1.5158	1.0640
19	0.8066	0.5544	1.3920	1.1284
20	0.9858	0.5069	1.2992	1.2897
21	1.2099	0.4277	1.0208	1.2252
22	1.0755	0.4911	1.2064	1.1607
23	0.9410	0.5148	1.2992	1.4186
24	0.8962	0.5069	1.2374	1.3541
25	1.0755	0.4990	1.1755	1.2897

The normalized decision values were multiplied by weight of the PMs obtained using MEREC method, to find the weighted normalized decision matrix, as shown in Table 8.

**Table 8:** Weighted normalized decision matrix.

Experiment no.	CO_2_ emission (%)	Heat generation (kW)	Methane emission (%)	Temperature of the trench (℃)
1	0.3383	0.0919	0.3294	0.2107
2	0.3045	0.0889	0.3378	0.1953
3	0.2537	0.0949	0.3631	0.1850
4	0.2368	0.0964	0.3716	0.1798
5	0.3214	0.0904	0.3547	0.1953
6	0.3045	0.0934	0.3462	0.1901
7	0.3552	0.0949	0.3547	0.2004
8	0.3890	0.0979	0.3800	0.2055
9	0.3045	0.1024	0.3631	0.2004
10	0.2199	0.1084	0.4054	0.1850
11	0.2875	0.0919	0.3547	0.1798
12	0.3552	0.0889	0.3294	0.2261
13	0.3214	0.1009	0.3631	0.2107
14	0.3383	0.1100	0.3969	0.1850
15	0.3552	0.1009	0.3969	0.1798
16	0.4229	0.0828	0.3125	0.2210
17	0.4060	0.0889	0.2956	0.2107
18	0.2706	0.1115	0.4138	0.1696
19	0.3045	0.1054	0.3800	0.1798
20	0.3721	0.0964	0.3547	0.2055
21	0.4567	0.0813	0.2787	0.1953
22	0.4060	0.0934	0.3294	0.1850
23	0.3552	0.0979	0.3547	0.2261
24	0.3383	0.0964	0.3378	0.2158
25	0.4060	0.0949	0.3209	0.2055

Next, the weighted proximity indices were computed from the ideal best values of each performance measure among all the twenty-five experimental runs. These values are given in Table 9.

**Table 9:** Weighted and overall proximity indices.

Experiment no.	CO_2_ emission (%)	Heat generation (kW)	Methane emission (%)	Temperature of the trench (℃)	Single performance score (OPI, ${d}_{i}$)
1	0.1184	0.0196	0.0844	0.0411	0.2635
2	0.0846	0.0226	0.0760	0.0257	0.2089
3	0.0338	0.0166	0.0507	0.0154	0.1165
4	0.0169	0.0151	0.0422	0.0103	0.0845
5	0.1015	0.0211	0.0591	0.0257	0.2074
6	0.0846	0.0181	0.0676	0.0206	0.1908
7	0.1353	0.0166	0.0591	0.0308	0.2418
8	0.1691	0.0136	0.0338	0.0360	0.2525
9	0.0846	0.0090	0.0507	0.0308	0.1751
10	0.0000	0.0030	0.0084	0.0154	0.0269
11	0.0677	0.0196	0.0591	0.0103	0.1566
12	0.1353	0.0226	0.0844	0.0565	0.2989
13	0.1015	0.0105	0.0507	0.0411	0.2038
14	0.1184	0.0015	0.0169	0.0154	0.1522
15	0.1353	0.0105	0.0169	0.0103	0.1730
16	0.2030	0.0286	0.1013	0.0514	0.3843
17	0.1861	0.0226	0.1182	0.0411	0.3680
18	0.0507	0.0000	0.0000	0.0000	0.0507
19	0.0846	0.0060	0.0338	0.0103	0.1347
20	0.1522	0.0151	0.0591	0.0360	0.2624
21	0.2368	0.0301	0.1351	0.0257	0.4277
22	0.1861	0.0181	0.0844	0.0154	0.3040
23	0.1353	0.0136	0.0591	0.0565	0.2645
24	0.1184	0.0151	0.0760	0.0462	0.2557
25	0.1861	0.0166	0.0929	0.0360	0.3315

### S/N ratio, ANOM and ANOVA

In this step, the S/N ratio was calculated from the single performance scores (i.e. proximity index values, ${d}_{i}$). Since lower values of ${d}_{i}$ indices indicate higher similarity with the ideal run, the S/N ratio was obtained, as shown in Table 10 by considering lower the better characteristic, as given in Eq. (13):

**Table 10:** S/N ratio of the single performance scores (${d}_{i}$) for different experimental runs.

Experiment no.	Single performance score (OPI, ${d}_{i}$)	S/N ratio (dB)
1	0.2635	11.58
2	0.2089	13.60
3	0.1165	18.67
4	0.0845	21.46
5	0.2074	13.66
6	0.1908	14.39
7	0.2418	12.33
8	0.2525	11.96
9	0.1751	15.13
10	0.0269	31.41
11	0.1566	16.10
12	0.2989	10.49
13	0.2038	13.81
14	0.1522	16.35
15	0.1730	15.24
16	0.3843	8.31
17	0.3680	8.68
18	0.0507	25.89
19	0.1347	17.41
20	0.2624	11.62
21	0.4277	7.38
22	0.3040	10.34
23	0.2645	11.55
24	0.2557	11.84
25	0.3315	9.59

13 $$\eta =-10\mathrm{log}\left[\frac{1}{m}\sum_{i=1}^{m}{d}_{i}^{2}\right].$$

It is revealed from the S/N values of Table 10, that among the twenty-five experiments conducted as per L_25_ orthogonal design, the experimental setting of tenth run yields the best performance results. In order to further optimize the performance parameters, the S/N ratios were further analyzed by performing ANOM and ANOVA analyses, and their results are listed in Tables 11 and 12, respectively.

**Table 11:** Response table of mean S/N ratio for combined performance index (${d}_{i}$).

Input parameter	Level-1	Level-2	Level-3	Level-4	Level-5	∆ = Max.–Min.	Rank
A	15.80	17.04	14.40	14.38	10.14	6.90	2
B	11.55	11.09	16.38	16.44	16.31	5.35	3
C	19.12	14.44	14.11	12.36	11.73	7.38	1
D	12.08	15.80	13.54	16.46	13.90	4.38	5
E	11.86	16.06	17.07	14.33	12.44	5.21	4

**Table 12:** ANOVA results for combined performance index (${d}_{i}$).

Source of variation	Sum of square (SS)	Degrees of freedom	Mean square (MS)	% contribution
A	135.39	4	33.85	18.73
B	153.87	4	38.47	21.29
C	167.88	4	41.97	23.23
D	62.91	4	15.73	8.70
E	101.01	4	25.25	13.97
Error	101.76	4	25.44	14.08
Total	722.82	24	–	

It can be inferred from the mean S/N ratio depicted in Table 11 that the third input parameter, viz. parameter *C* (inorganic content in waste) has the highest influence on the multiple PMs of the landfill operations, followed by parameters *A*, *B*, *E* and *D*. Beside this, the optimal combination of input parameters can be obtained corresponding to the levels at which the S/N ratios are highest among all the levels. Hence, the optimal combination derived for the present case is *A*_2_*B*_4_*C*_1_*D*_4_*E*_3_, that is when: waste particle size (*A*) is 9 ppm, waste addition (*B*) is 80 Ktoe, inorganic content in waste (*C*) is 2%, leachate recirculation rate (*D*) is 250 *l*/day, and landfill age (*E*) is 3 years.

The results of ANOVA are shown in Table 12 which indicate that input parameter *C* is the most significant parameter for affecting the overall performance of landfill operation. Other input parameters in decreasing order of their significance are *B*, A, *E* and *D*. It also appears from Table 12 that the percentage contribution of error is slightly higher, i.e. 14%. This might be due to heavy metals present in small proportions in the form of boron, arsenic, selenium, lithium. This has changed the actual composition of the solid waste which might have contributed to the error making it slightly higher error. Furthermore, soil uncertainty in landfills presents both spatial variability and temporal variability. The inorganic content in the landfills also varies from waste to waste, thereby varying the decomposition process rate, leading to slightly uncertain outputs. However, considering the complexity of treating leachate in an economic and sustainable manner, the recirculation process seems to be a feasible option and the above error rate for invariable composition of waste is acceptable for the current set of experimental system. A high error rate may be tolerated in the implementation of leachate recirculation programs due to the complexity of the processes involved, the lack of reliable data and information, or resource constraints. For example, it can be challenging to accurately predict the volume and composition of leachate produced in landfills. In such scenarios, waste management programs may choose to adopt a pragmatic approach, recognizing the limitations of their methods and accepting a higher error rate in order to make progress in managing the waste effectively. However, it is important for these programs to continuously seek ways to improve their methods and reduce the error rate in order to provide more accurate and effective management of the leachate recirculation.

## Discussion

Leachate management within landfills is a complex domain due to the nature of the flow equations in unsaturated porous media with nonlinear inter-relationship among leachate characteristics. Apparently, researchers have found it challenging to find a sustainable solution for leachate management in landfills. The physicochemical properties of leachate can also pose difficulties in determining the values of unsaturated hydraulic conductivity in municipal solid waste landfills^36^. This highlights the importance of recirculating the leachate rather than treating it, as treatment can become even more expensive. Henceforth, the results drawn from the analysis will aid in contemplating the use of leachate recirculation process within landfills for increasing the cost-effectiveness and sustainability of the plant.

The density of the waste deposited in a landfill holds a vital significance in the leachate recirculation process. When the waste is compacted too tightly, the flow of leachate from the trench into the collection pipes is restricted, resulting in the formation of heat pockets at various locations within the landfill^37^. These heat pockets have been identified as the primary cause of landfill fires, which can cause harm to the landfill and release toxic methane into the environment through landslides. Landfill fires are challenging to control and extinguish, as the compacted waste and limited access make it difficult to reach the core of the fire and apply effective firefighting methods. Furthermore, smaller waste particles offer a greater surface area for chemical reactions, leading to a stabilized reaction rate and uniform heat generation during exothermic reactions^38^. This increased surface area allows for more contact points between waste materials and can enhance the rates of chemical reactions. Studies have proven that the thermal energy trapped in landfills is inversely proportional to the size of the waste particles^39^. With more surface area available, more waste particles can interact with oxygen and other reactive agents, facilitating a continuous heat-generating reaction. With smaller waste particle size, the concentration of methane increases, and the CO_2_ content decreases after degradation of organic waste.

In addition, the smaller particle size of waste influences the mechanical and hydraulic properties of the compacted waste, which can be regulated by controlling the recirculation of the collected cold leachate into the landfill. Higher surface area allows for increased bacterial decomposition activity, resulting in a rise in the internal temperature of the landfill^40^. In terms of mechanical properties, the smaller particles can interlock more effectively, creating a denser and more stable waste matrix. This increased interlocking can enhance the load-bearing capacity of the waste, making the landfill more resistant to settlement and deformation over time. Landfills with a higher shredding capacity and smaller waste particles have been found to exhibit improved settlement and stability, resulting in fewer voids for heat entrapment. Settlement refers to the gradual sinking of the landfill over time due to the compression of waste materials. Minimizing settlement is essential to maintain the landfill’s structural integrity and prevent damage to the waste containment system. Moreover, increased biological activity leads to an improved compression index of waste, enabling the accommodation of more waste in the same area^41^. In landfills with larger waste particles and more significant void spaces, heat might be confined and concentrated, potentially leading to the formation of heat pockets. The movement and distribution of leachate within a landfill is dictated by the hydraulic conductivity of the MSW. This value can vary greatly depending on factors such as the waste composition, compaction, pressure, and distribution of pore sizes. Increased biological activity results in more efficient decomposition of organic waste, causing it to break down and compress further. In the context of landfills, hydraulic conductivity measures how easily water or leachate can flow through the waste mass. Additionally, hydraulic conductivity in landfills can also vary over time as the organic matter degradation process progresses, leading to a decrease in particle size and an increase in the specific weight of the residue, ultimately resulting in lower values of hydraulic conductivity^42^. As the proportion of non-degradable components increases in the residue, the specific weight of the landfill waste also increases.

The presence of inorganic substances in a landfill greatly impacts the generation of leachate generation capacity. The presence of inorganic substances in the landfill significantly impacts the leachate generation capacity and poses challenges for its proper handling. In a landfill, the quantity of inorganic substances plays a significant role in the generation and management of leachate. When inorganic substances are present in large quantities, the leachate generation capacity increases, making it more difficult to pump and manage the fluid. Since inorganic substances do not undergo biological decomposition, so they remain intact in the landfill for extended periods. This leads to a reduced rate of waste degradation overall, which affects the amount of leachate generated. To mitigate this fluid, proper segregation techniques should be implemented to remove substances such as plastics, woods, glass, and metals, and increase the percentage of organic matter in the landfill^43^. This separation can significantly reduce the presence of inorganic substances in areas where leachate is generated, helping to control the leachate volume. Reducing the inorganic matter in waste resources has the added benefit of increasing the methane content. Inorganic substances, including surplus CO_2_, can slow down the chemical reaction necessary for methane generation. Surplus carbon dioxide (CO_2_) in the waste stream can also inhibit the formation of methane. Excessive CO_2_ can limit the availability of hydrogen for methane production, leading to a lower methane content in the biogas. Heavy metals are often present in various waste streams, particularly electronic waste and certain plastics. During the waste reclamation process, these heavy metals can be released and concentrated in the syngas. This increases the concentration of heavy metals in the syngas, making the recovery of methane expensive and challenging. As a result, it's critical to properly separate inorganic waste from biodegradable waste in waste reclamation facilities^44^. Inorganic substances emit more carbon than organic substances, resulting in a higher CO_2_ content in waste that contains a higher quantity of inorganic matter. By removing inorganic substances from the waste stream, the hindrance to methane generation can be reduced, resulting in a higher methane content in the produced biogas. Therefore, effective management and segregation of inorganic substances in landfills is crucial for a successful leachate recirculation process and sustainable waste management practices. By paying close attention to these details, the negative impacts of inorganic waste can be minimized.

The efficient management of leachate in a landfill is crucial for the successful operation of the site. The recirculation rate of leachate has a direct impact on the heat generation capacity of the landfill, with an increase in recirculation leading to an increase in heat generation up to a certain point. The recirculated leachate comes into contact with the decomposing waste, providing additional moisture and nutrients to support biological activity. However, if the recirculation rate exceeds that point, it may cause flooding and obstruct the heat generation process. This leads to an increase in the heat generation capacity of the landfill. Effective leachate recirculation provides the necessary nutrients for the thermal decomposition reaction, leading to an increase in methane concentration and a decrease in CO_2_ content. Previous studies have shown a 10% increase in methane production with the implementation of a proper leachate recirculation process^45^. The presence of these nutrients and microorganisms supports the process of thermal decomposition, which is the breakdown of organic waste through microbial activity in the presence of heat. During monsoon seasons, it is important to store the excess leachate in a facility in order to maintain the optimum humidity conditions in the landfill trench. The temperature rise in the landfill decreases with an increase in leachate recirculation, as the spread of cold leachate helps to reduce the surface temperature of the decomposed waste^46^. However, the increased flow rate of leachate may also cause partial clogging of the water collection system, leading to a decrease in hydraulic conductivity of the drainage film and solidifying the leachate at the bottom of the clay liner. Studies have shown that the optimum temperature for methane generation is between 25 and 30 ℃, with a linear decrease in gas generation recorded at temperatures above 55 ℃. To ensure that the internal temperatures in the landfill do not exceed 70 ℃, it is essential to implement a leachate recirculation facility that can monitor and control the heat release rate^47^. However, if the flow rate of leachate becomes too high, it can overwhelm the water collection system and result in partial clogging, reducing its effectiveness in draining excess water. Table 13 demonstrates the validation of the current model by showcasing above par methane generation results compared to previous leachate recirculation techniques found in prior studies. This validation solidifies the justification for the effectiveness of the present model.

**Table 13:** Validation of model with prior available analysis for methane generation.

S. no	Reference	Model	Methane generation (%)
1	Zhang et al.^39^	Conventional	40
2	Mehdard et al.^50^	Support vector machine	45
3	Current study	MEREC-PIV	48

The composition of leachate is greatly influenced by the age of the waste in a landfill. The interaction between leachate and waste can modify the makeup of the leachate, resulting in varying levels of certain elements such as boron, arsenic, selenium, and lithium. The concentration of key inorganic components, such as calcium, magnesium, sodium, potassium, iron, and sulphates, is determined by the stabilization processes within the waste mass^48^. The thermal energy generated by a landfill undergoes a linear increase in the first three years after it has been closed, peaking around the 10th or 15th year, when the waste's biodegradation capacity is exhausted. However, with the implementation of a temperature control system, through recirculating leachate, the stabilization time is greatly reduced, leading to an increase in methane emission concentration before natural stability is attained^49^. The implementation of this system also results in earlier peak methane production, occurring in the fourth year, and a quicker ability for landfill investors to capture syngas, leading to substantial profits and long-term environmental and energy benefits, including the addition of carbon credits, starting in the third year.

The current study’s approach of utilizing optimal waste characteristics and leachate recirculation, represented by the MEREC-PIV model, demonstrated superior results in methane generation compared to prior research. The conventional model used by Zhang et al.^39^ yielded 40% methane generation, while Mehdard et al.^50^ employed a Support Vector Machine model resulting in 45% methane generation. In contrast, the current study achieved a significant improvement, reaching 48% methane generation. This indicates that the utilization of MEREC-PIV, combined with the optimized waste characteristics and leachate recirculation, outperformed both conventional and Support Vector Machine models in enhancing methane production, showcasing the superiority and effectiveness of the current approach.

## Conclusions

Traditional landfill management practices often result in a substantial amount of leachate generation that must be transported to external wastewater treatment facilities, which are typically located far from the landfill site. However, by reintroducing the leachate into the landfill under optimal conditions and treating it as a managed bioreactor, both economic and environmental benefits can be availed. The present study attempted to optimize the main input parameters which are responsible for improved leachate regeneration rate and consequently for sustainable performance of landfills. The influence of five input parameters on the physio-chemical characteristics of the landfill was investigated using Taguchi’s L_25_ orthogonal array. The PMs of the landfill were defined and obtained in terms of four output variables. Multi-performance optimization was carried out using integrated MEREC-PIV methods followed by Taguchi’s approach comprising of ANOM and ANOVA. The following conclusions are drawn from the present study:

- Among the four performance measures, CO_2_ emission is the most important one followed by methane emission, heat generation and temperature of the trench.
- The optimal combination of the input parameters for optimum multiple performance measures of the landfill operations is: waste particle size = 9 ppm, waste addition = 80 Ktoe, inorganic content in waste = 2%, leachate recirculation rate = 250 l/day and landfill age = 3 years.
- The inorganic content in waste is the most influential input parameter that affects the multiple performance measures of the landfill operations. Other input parameters in the order of their decreasing significance are waste addition, waste particle size, landfill age and leachate recirculation rate.

## Current limitations and future research scopes

Although the present work discloses a way for sustainable alternative to costly treatment processes, there are several limitations associated with the study due to various constraints including resource constraint. Firstly, the recirculating leachate can be a complex process that requires specialized equipment, pumps, and piping. This can be challenging to install and maintain, especially in older landfills. Secondly, the leachate is often highly acidic, which can cause corrosion of the equipment and pipes used in the recirculation process. Thirdly, the implementation and maintenance of leachate recirculation system can be expensive, especially in older landfills where retrofitting may be necessary. Finally, the recirculation can help control leachate levels and reduce the amount of leachate that needs to be treated and disposed, though, it may not solve all leachate management problems, thereby requiring additional treatment methods.

There are also a few limitations associated with the present model for finding the optimal input parameters, discussed as follows, which can be considered in the future research work. The accurate prediction of the timing and the quantity of gas and leachate production in bioreactor landfills is a complex task, and it's essential to specify the potential limitations of the models used for such predictions. The variability in waste composition within landfills can significantly impact decomposition rates and, consequently, gas and leachate production. The current model has assumed uniform waste composition. Moreover, gas and leachate production rates can vary over time due to factors like waste compaction, changing moisture levels, and the age of waste, which have not been adequately accounted for in the present study. Furthermore, the heterogeneous nature of waste decomposition rates poses a challenge, as different materials decompose at different rates, and models have used an average degradation rate of 0.035 per year, which does not reflect the true landfill composition. Additionally, the complex microbial processes responsible for waste decomposition are influenced by factors like temperature, pH, and nutrient availability, and this necessitates their incorporation for higher model accuracy. Each landfill site has unique characteristics that affect gas and leachate production, including topography, landfill design, and operational practices. These site-specific factors may not be fully integrated into models. Finally, the availability of accurate and up-to-date data is essential for model accuracy but may not always be readily available or may be subject to measurement errors.

Apart from this, the performance of the landfill operation can also be assessed using different MCDM techniques. A comparative analysis can be carried out to find the most suitable technique for assessment.

## Data Availability

All data supporting the findings of this research are included in the paper.
